# Bergamottin Inhibits PRRSV Replication by Blocking Viral Non-Structural Proteins Expression and Viral RNA Synthesis

**DOI:** 10.3390/v15061367

**Published:** 2023-06-13

**Authors:** Zhenbang Zhu, Yuqian Xu, Lulu Chen, Meng Zhang, Xiangdong Li

**Affiliations:** 1 Jiangsu Co-Innovation Center for Prevention and Control of Important Animal Infectious Diseases and Zoonoses, College of Veterinary Medicine, Yangzhou University, Yangzhou 225009, China; 007583@yzu.edu.cn (Z.Z.); xuyuqian_dina@163.com (Y.X.); mz120170987@yzu.edu.cn (L.C.); zm17852410578@163.com (M.Z.); 2 Joint International Research Laboratory of Agriculture and Agri-Product Safety, The Ministry of Education of China, Yangzhou University, Yangzhou 225009, China

**Keywords:** PRRSV, begamottin, non-structural protein, cytokines, antiviral activity

## Abstract

The porcine reproductive and respiratory syndrome virus (PRRSV) causes economic losses in the swine industry worldwide. However, current vaccines cannot provide effective protection against PRRSV, and PRRSV-specific treatments for infected herds are still unavailable. In this study, we found that bergamottin showed strong inhibitory effects against PRRSV replication. Bergamottin inhibited PRRSV at the stage of the replication cycle. Mechanically, bergamottin promoted the activation of IRF3 and NF-κB signaling, leading to the increased expression of proinflammatory cytokines and interferon, which inhibited viral replication to some extent. In addition, bergamottion could reduce the expression of the non-structural proteins (Nsps), leading to the interruption of replication and transcription complex (RTC) formation and viral dsRNA synthesis, ultimately restraining PRRSV replication. Our study identified that bergamottin possesses potential value as an antiviral agent against PRRSV in vitro.

## 1. Introduction

The porcine reproductive and respiratory syndrome virus (PRRSV) is one of the most important pathogens in the global swine industry, which causes huge economic losses all around the world [1,2]. Porcine reproductive and respiratory syndrome (PRRS), caused by PRRSV, is characterized by serious reproductive disorders in sows and respiratory symptoms in piglets with high mortality [3]. PRRSV is a positive-strand RNA virus with an almost 15 kb genome size. The PRRSV genome possesses at least ten ORFs, with genes encoding viral replicase and genes encoding structural proteins. PRRSV replicase genes consist of two ORFs, ORF1a and ORF1b, which encode at least 14 functional non-structural proteins (Nsps) [4,5]. Most Nsps are relevant to viral replication, virulence, and host immunosuppression. A network of viral replicase proteins and some cellular proteins are the major components of the replication and transcription complexes (RTCs) [6]. RTC assembly is responsible for the synthesis of viral RNA, which coordinates the transcription and replication cascade. PRRSV Nsp2, Nsp3, and Nsp5 are the scaffolding components of RTCs [7]. Moreover, Nsp9, Nsp10, Nsp11, and Nsp12 are the core components that are recruited by these membrane scaffolding proteins to the replicase sites [8]. Among these Nsps, PRRSV Nsp10 functions as an RNA helicase, unwinding RNA secondary structures and benefiting sub-genomic mRNA (sg mRNA) synthesis [9]. PRRSV Nsp10 is also reported as the key virulence gene of HP-PRRSV and plays an important role in virus replication [10]. In addition, other Nsps are responsible for PRRSV replication, immunosuppression, immune evasion, and some underlying pathogenicity [11]. To prevent PRRSV infection, many anti-PRRSV strategies and techniques have been developed. Vaccination is still a common approach to control PRRSV infection, although it has achieved only limited success [12]. Notably, herbal extracts and some chemical compounds in recent research have shown powerful inhibitory effects on PRRSV infection; however, there is a long way to go for practical use in anti-PRRSV therapy [13].

Furanocoumarins are extracted from the pulp of grapefruits (grapefruit juice), which are the secondary metabolites produced in citrus fruits [14]. Bergamottin (BGM) is one of the important natural derivatives of furanocoumarin, which contributes to protecting plants from pathogens [15]. In recent studies, bergamottin has been identified as a producer of anti-cancer activity and has become a potential candidate as a dietary supplement against diverse cancers, including myeloma, melanoma, prostate cancer, and so on [16,17,18]. Meanwhile, bergamottin can activate different signaling pathways to regulate the vital activities of cells [19,20,21]. Recent research has demonstrated that bergamottin has an influence on virus infection. Bergamottin can inhibit SARS-CoV-2 infection by inducing ACE2 degradation [22,23]. Bergamottin suppresses Lassa virus (LASV) entry by blocking endocytosis and trafficking [24]. Bergamottin also shows inhibitory effects on the authentic lymphocytic choriomeningitis virus [24]. Whether bergamottin has an influence on PRRSV replication is unclear.

In this study, we found that bergamottin has strong antiviral activity against PRRSV infection. Mechanically, bergamottin inhibited the initial expression of most Nsps, thus blocking viral RNA synthesis and PRRSV replication. These results provide a novel experimental way of blocking PRRSV replication.

## 2. Materials and Methods

### 2.1. Cells, Viruses, and Bergamottin

Marc-145 cells, derived from African green monkey kidney cells, allow for PRRSV replication. Marc-145 cells were cultured in Dulbecco’s modified Eagle’s medium (DMEM) (Gibco, Waltham, MA, USA) containing 10% fetal bovine serum (FBS). Porcine alveolar macrophages (PAMs), the target cells for PRRSV infection, were isolated from broncho-alveolar lavage fluid of piglets and were maintained in RPMI 1640 medium with 10% FBS at 37 °C in 5% CO_2_. Four PRRSV strains (CHR6, SD16, XJ17-5, and Li11) were used in this study. The CHR6 strain was used in most of our assays. SD16, XJ17-5, and Li11 strains were used to identify the antiviral effects on different PRRSV strains. CHR6 and Li11 were kept in our laboratory, and SD16 and XJ17-5 were provided by Prof. Nanhua Chen from Yangzhou University. CHR6 and SD16 belong to the classical strain, while XJ17-5 and Li11 strains are highly pathogenic PRRSV strains. All PRRSV titers were determined as 50% tissue culture infective dose (TCID_50_) on Marc-145 cells. Bergamottin was available commercially (MCE, Princeton, NJ, USA). It was diluted in dimethylsulfoxide (DMSO) at a concentration of 10 mM, stored at 4 °C, and protected from light.

### 2.2. Cytotoxicity Assay

Cell viability after treatment with bergamottin was measured using the Enhanced Cell Counting Kit-8 (CCK-8; Beyotime, Shanghai, China). Marc-145 cells or PAMs were seeded in 96-well plates (1 × 10^4^ cells/well). Then, different concentrations of bergamottin were added to the cells and incubated for 36 or 48 h at 37 °C, followed by incubation with 10 μL CCK-8 solution per well for another 3 h. The absorbance was measured at 450 nm, which indicated the relative cytotoxicity of bergamottin.

### 2.3. Quantitative Real-Time Reverse-Transcription Polymerase Chain Reaction (RT-qPCR)

The mRNA expression of PRRSV N, IL-1β, IL-6, IFN-α, and IFN-β was detected with RT-qPCR. Cells were collected, and total mRNA was extracted using TRIzol reagent (TIANGEN, Beijing, China). A total of 1 μg mRNA was inverse-transcribed into cDNA using HiScript IIIRT SuperMix (Vazyme, Nanjing, China), according to the manufacturer’s instructions. The cDNA products were amplified with indicated primers and a ChamQ Universal SYBR qPCR Master Mix (Vazyme, Nanjing, China) for gene quantitative PCR (qPCR) on ABI QuantStudio3 (Applied Biosystems, Waltham, MA, United States). The primers are listed below: PRRSV N-F: AAAACCAGTCCAGAGGCAAG; PRRSV N-R: CGGATCAGACGCACAGTATG; IL-6-F: AGAGGCACTGGCAGAAAAC; IL-6-R: TGCAGGAACTGGATCAGGAC; IL-1β-F: GGAAGACAAATTGCATGG; IL-1β-R: CCCAACTGGTACATCAGCAC; IFN-β-F: GCAATTGAATGGAAGGCTTGA; IFN-β-R: CAGCGTCCTCCTTCTGGAACT; IFN-α-F: TCCAGCTCTTCAGCACAGAG; IFN-α-R: AGCTGCTGATCCAGTCCAGT; GAPDH-F: TGACAACAGCCTCAAGATCG; and GAPDH-R: GTCTTCTGGGTGGCAGTGAT. The qPCR reaction was performed as follows: 95 °C for 1 min, followed by 40 cycles at 95 °C for 5 s and 60 °C for 1 min. The data were calculated using the 2^−ΔΔCt^ method and normalized to GAPDH. Three replicates were included for each treatment.

### 2.4. Western Blot

When cells were treated with PRRSV or bergamottin for the indicated durations, cells were washed with PBS and harvested with a cell lysis buffer (Beyotime, Shanghai, China) on ice for at least 20 min. Whole-cell lysates were quantified using the BCA method (Beyotime, Shanghai, China). Quantified protein lysates were subjected to 10–12% sodium dodecyl sulfate-polyacrylamide gel electrophoresis (SDS-PAGE) and transferred to a polyvinyl difluoride (PVDF) membrane (Merck Millipore, Billerica, MA, USA). After the electro-transfer, membranes were blocked with 5% skim milk (Sangon Biotech, Shanghai, China) in TBST (20 mM Tris-HCl PH8.0, 150 mM NaCl, 0.05% Tween 20) at room temperature for 1 h. Then, the membranes were cropped and incubated with indicated primary antibodies, including anti-PRRSV N (MEDIAN, Republic of Korea) antibody, anti-mCherry (Abcam, Cambridge, UK) antibody, and anti-GAPDH, p65, phosphorylated p65, IRF3, phosphorylated IRF3, phosphorylated IκBα (Cell Signaling Technology, Danvers, MA, USA) antibodies at a dilution of 1:1000 at 4 °C overnight. Among them, GAPDH served as an internal control. After rinsing with PBS three times, membranes were incubated with corresponding secondary antibodies at 1:5000 for 1 h. Finally, signals were visualized using an enhanced chemiluminescence reagent (NCM Biotech, Suzhou, China) on a Tanon 5200 system (Tanon, Shanghai, China).

### 2.5. Expression Vector Construction and Transfection

PRRSV Nsp1, Nsp2, Nsp4, Nsp5, Nsp7, Nsp8, Nsp10, Nsp11, and Nsp12 genes were amplified from the PRRSV CHR6 strain and subcloned into pmCherryN1 Vector (632523; Takara, Japan) with an N-terminal mCherry tag. HEK293T cells were seeded in six-well plates (2 × 10^6^ cells/well), and the cells were transfected with these mCherry-tagged Nsps plasmids. The final concentration of each plasmid was 3000 ng, and Lipofectamine 2000 (Invitrogen, Carlsbad, CA, USA) was used for plasmid transfection. The cells were transfected with these plasmids for 24 h in the absence or presence of bergamottin and finally collected for immunofluorescence analysis and western blot.

### 2.6. Viral Binding, Entry, Replication, and Release Assays

For the binding assay, Marc-145 cells were precooled at 4 °C for 1 h and then infected with PRRSV (MOI = 5) in the presence of different concentrations of bergamottin (0, 12.5, 25, and 50 μM) for 2 h at 4 °C, allowing viral particles to bind to cell surfaces but not to be internalized. Cells were washed with cold PBS three times, and bound virions were measured by RT-qPCR. Three replicates were included for each treatment.

For the entry assay, Marc-145 cells were infected with PRRSV (MOI = 5) for 2 h at 4 °C. After rinsing with cold PBS three times, the cells were treated with different concentrations of bergamottin (0, 12.5, 25, and 50 μM) and incubated for another 2 h at 37 °C. Then, an alkaline high-salt solution [1 M NaCl and 50 mM sodium bicarbonate (pH 9.5)] was used to remove cell surface–associated viruses [25]. Intracellular PRRSV RNA was detected using RT-qPCR. Three replicates were included for each treatment.

For the replication assay, Marc-145 cells were infected with PRRSV (MOI = 1) for 4 h and then treated with bergamottin (25 μM) for another 44 h. Cells were collected for RT-qPCR at 12, 24, 36, and 48 hpi.

For the release assay, Marc-145 cells were infected with PRRSV (MOI = 1) for 24 h. Then, the cells were washed with PBS and treated with different concentrations of bergamottin (0, 12.5, 25, and 50 μM) for another 12 h, at which time the viruses were released from the cells. Cell supernatants were collected for TCID_50_.

### 2.7. Immunofluorescence Assay

After the treatments with PRRSV and bergamottin, the cells were fixed with paraformaldehyde (Biosharp, China) for 10 min and permeabilized with 0.5% Triton X-100 for 15 min. Then, the cells were blocked with 3% BSA in PBST for 30 min, followed by incubation with primary antibodies at 4 °C overnight. The antibodies included an anti-NF-κB p65 antibody (Cell Signaling Technology, Danvers, MA, USA) and an anti-dsRNA antibody (Scicons, Szirák, Hungary). After washing with PBST three times, the cells were incubated with indicated secondary antibodies for 1 h. Then, the cells were washed with PBST three times and counterstained with 4′, 6-diamidino-2-phenylindole dihydrochloride (DAPI) (Beyotime, Shanghai, China) in PBS for an additional 5 min. The stained cells were visualized using a confocal laser scanning microscope (TCS SP8 STED; LEICA, Weztlar, Germany) or an inverted fluorescence microscope (UHGLGPS; OLYMPUS, Tokyo, Japan).

### 2.8. Detection of Proinflammatory Cytokines and Interferon

To evaluate the effect of bergamottin on proinflammatory cytokines and interferon, Marc-145 cells were mock-infected or infected with PRRSV (MOI = 1) in the presence (25 μM) or absence of bergamottin. Cells were collected at 12, 24, and 36 hpi. RT-qPCR was used to measure the mRNA relative expression of IL-1β, IL-6, IFN-α, and IFN-β. GAPDH served as an internal reference. Three replicates were included for each treatment.

### 2.9. Statistical Analysis

All experiments were performed with at least three independent replicates. Statistical analysis was performed using SPSS 17.0 and GraphPad Prism 5.0. Data are presented as mean ± SEM. The student’s *t*-test and one-way ANOVA were used to analyze the data. *p* < 0.05 was considered to be significant.

## 3. Results

### 3.1. Bergamottin Inhibits PRRSV Replication

**Figure 1:**
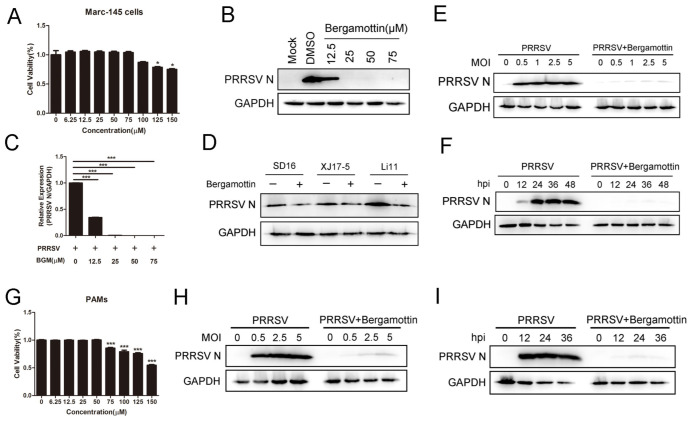
Bergamottin effectively restrained PRRSV replication in both Marc-145 cells and PAMs. (**A**) The cytotoxicity of bergamottin in Marc-145 cells was measured using a CCK-8 assay. Marc-145 cells were treated with bergamottin at indicated concentrations for 48 h, and cell viability assay was performed. (**B**) Marc-145 cells were mock-infected or infected with PRRSV (MOI = 1) in the presence of different concentrations of bergamottin (0, 12.5, 25, 50, 75 μM). Viral N protein was detected using western blot. (**C**) Marc-145 cells were mock-infected or infected with PRRSV (MOI = 1) in the presence of different concentrations of bergamottin (0, 12.5, 25, 50, 75 μM). The mRNA expression of PRRSV N was detected using RT-qPCR. (**D**) Marc-145 cells were infected with different PRRSV strains (SD16, XJ17-5, and Li11; MOI = 1) in the absence or presence of bergamottin (25 μM); western blot was used to detect the expression of PRRSV N protein. (**E**) Marc-145 cells were infected with PRRSV at different MOIs in the presence (25 μM) or absence of bergamottin. N protein was determined using western blot analysis. (**F**) Marc-145 cells were infected PRRSV (MOI = 1) for indicated durations (0, 12, 24, 36, and 48 hpi) in the absence or presence of bergamottin (25 μM). Western blot showed the expression of PRRSV N protein. (**G**) The cytotoxicity of bergamottin in PAMs was measured using a CCK-8 assay. (**H**,**I**) The same treatments were performed in PAMs (bergamottin (25 μM)), and western blot was used to analyze the viral N protein at different MOIs (**H**) and different infection periods (**I**). Data are the results of three independent experiments (means ± SE). Significant differences are denoted by * *p* < 0.05, and *** *p* < 0.001.

Bergamottin is a bioactive component of bergamot, which protects plants from pathogens. We first investigated whether bergamottin showed antiviral activity against PRRSV replication on Marc-145 cells. Marc-145 cells cultured in a medium containing 75 μM bergamottin retained approximately relative viability of 100% compared with controls after treatment for 48 h (Figure 1A). Marc-145 cells were mock-infected or infected with PRRSV CHR6 (MOI = 1) in the presence of different concentrations of bergamottin (12.5, 25, 50, and 75 μM) for 24 h. As shown in Figure 1B, bergamottin reduced PRRSV N protein expression and completely inhibited PRRSV replication at 25 μM. Similarly, the mRNA level of viral ORF7 was significantly decreased in a dose-dependent manner when compared with the control group (Figure 1C). Next, we identified the antiviral effects of bergamottin on different strains. The results showed that bergamottin could restrain both the PRRSV classical strain (SD16) and HP-PRRSV (XJ17-5 and Li11). However, compared with the CHR6 strain, it showed only minor inhibition of other PRRSV strain replications (Figure 1D). Marc-145 cells were mock-treated or treated with bergamottin and then infected with different MOIs of PRRSV. PRRSV N expression was inhibited when the cells were treated with bergamottin regardless of the amount of virus (Figure 1E). Moreover, Marc-145 cells were mock-treated or treated with bergamottin and then infected with PRRSV for different durations. As shown in Figure 1F, PRRSV N protein was obviously reduced in the presence of bergamottin compared to the control group. To further investigate the antiviral effects on PRRSV, we carried out the same experiment on porcine alveolar macrophages (PAMs). Compared to the mock-treated cells, cell viability still reached 100% at a concentration of 50 μM (Figure 1G). Western blot showed that bergamottin inhibited PRRSV replication in time- and MOI-dependent on PAMs (Figure 1H,I). Altogether, bergamottin had the potential to suppress PRRSV replication.

### 3.2. Bergamottin Blocks PRRSV Entry and Genome Replication but Had No Effects on Viral Binding or Release

**Figure 2:**
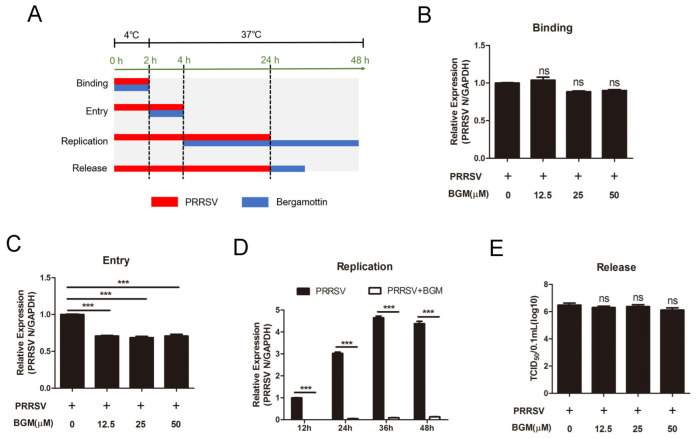
Bergamottin inhibited PRRSV infection at the stage of entry and replication. (**A**) Schematic diagram of viral attachment, entry, replication, and release. For binding assay, Marc-145 cells were precooled at 4 °C for 1 h and then inoculated with PRRSV (MOI = 5) in the presence or absence of bergamottin for 2 h at 4 °C. For entry assay, after viral binding, cells were washed with cold PBS and treated with bergamottin; then, cells were transferred to 37 °C for another 2 h. For replication assay, cells were infected with PRRSV (MOI = 1) for 4 h and then treated with bergamottin for another 44 h. For release assay, Marc-145 cells were infected with PRRSV (MOI = 1) for 24 h; then, cells were washed with PBS and treated with bergamottin for another 12 h. (**B**) Viral binding assay was performed, and RT-qPCR was used to detect PRRSV N expression. (**C**) Viral entry assay was performed; RT-qPCR was used to analyze viral internalization. (**D**) Viral replication assay was performed; cells were collected at 12, 24, 36, and 48 hpi for RT-qPCR analysis. (**E**) Viral release assay was performed; cell supernatants were collected for TCID_50_. Data are the results of three independent experiments (means ± SE). Significant differences are denoted by *** *p* < 0.001.

To further investigate the viral life cycle that was blocked by bergamottin, a binding assay, entry assay, replication assay, and release assay were performed (Figure 2A). For the binding assay, cells were incubated with PRRSV and different concentrations of bergamottin at 4 °C for 2 h to permit virus attachment. There was no significant difference in the stage of viral binding (Figure 2B). After the attachment assay, cells were transferred to 37 °C for another 2 h allowing virus entry. As shown in Figure 2C, bergamottin significantly decreased PRRSV internalization, showing a reduction in PRRSV ORF7 mRNA expression. Meanwhile, compared with only PRRSV treatment, the mRNA level of PRRSV N was remarkably reduced in the presence of bergamottin during the PRRSV replication stage (Figure 2D). For the release assay, different concentration of bergamottin was added at 24 hpi for another 4 h; cell supernatant was collected for TCID_50_. There was no significant difference in PRRSV release in the absence or presence of bergamottin (Figure 2E). These results indicated that bergamottin blocked PRRSV internalization and replication but had little influence on viral attachment and release.

### 3.3. Bergamottin Activates IRF3 and NF-κB Signaling Pathway

**Figure 3:**
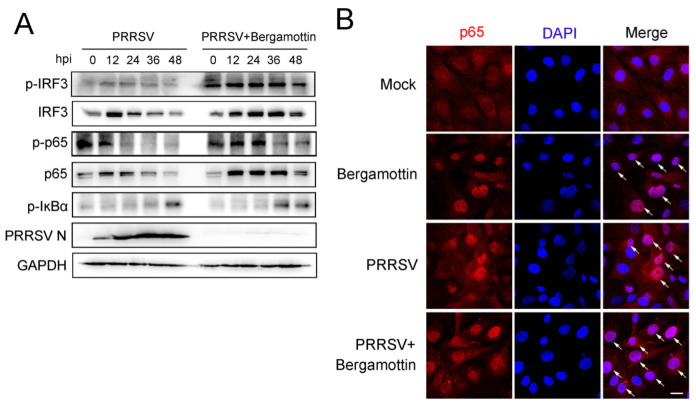
Bergamottin facilitated the activation of IRF3 and NF-κB. (**A**) Marc-145 cells were infected with PRRSV (MOI = 1) in the presence (25 μM) or absence of bergamottin. Cells were collected at 0, 12, 24, 36, and 48 hpi for western blot analysis. Phosphorylated IRF3, phosphorylated p65, phosphorylated IκBα, total IRF3, and total p65 were detected. GAPDH is shown as an internal reference. (**B**) Marc-145 were mock-infected or infected with PRRSV (MOI = 1) for 24 h in the presence (25 μM) or absence of bergamottin. Cells were fixed and stained for NF-κB p65 antibody overnight. Then, cells were incubated with an Alexa fluor 555-conjugated anti-rabbit IgG (red). Nuclei were counterstained with DAPI (blue). White arrows represent p65 nuclear translocation. Bar = 25 μm.

In order to identify the signaling pathways by which bergamottin inhibits PRRSV replication, Marc-145 cells were mock-treated or treated with bergamottin, and then cells were infected with PRRSV for different durations. As shown in Figure 3A, the phosphorylation of IRF3 was increased to a high level when cells were treated with bergamottin. Simultaneously, phosphorylated NF-κB p65 was enhanced at 24, 36, and 48 hpi in the presence of bergamottin when compared to those in only PRRSV-treated cells. The phosphorylation of IκBα was also up-regulated at 36 hpi in bergamottin-treated cells (Figure 3A). These data suggested bergamottin can activate IRF3 and NF-κB pathways. The activation of the NF-κB pathway requires NF-κB p65 phosphorylation and nuclear translocation. To further demonstrate NF-κB signaling participation in the antiviral activity of bergamottin, immunofluorescence analysis was performed. Compared with the mock-treated cells, bergamottin facilitated NF-κB p65 translocation from the cytoplasm to the nucleus. When Marc-145 cells were infected with PRRSV, the NF-κB pathway was activated, and p65 was translocated into the nucleus in some of the cells. Once Marc-145 cells were treated with bergamottin, p65 nuclear translocation occurred in almost all cells compared to the cells treated with PRRSV alone (Figure 3B). These data indicated that bergamottin can stimulate IRF3 and NF-κB pathways.

### 3.4. Bergamottin Promotes the Expression of Proinflammatory Cytokines and Type I Interferon

**Figure 4:**
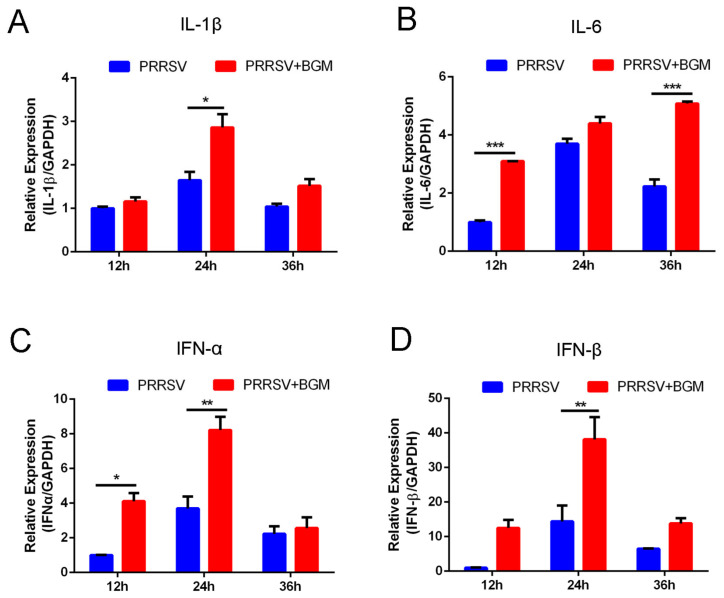
Bergamottin promoted the expression of proinflammatory cytokines and interferon. Marc-145 cells were mock-infected or infected with PRRSV (MOI = 1) in the presence (25 μM) or absence of bergamottin. RT-qPCR was used to measure the mRNA expression of IL-1β (**A**), IL-6 (**B**), IFN-α (**C**), and IFN-β (**D**) at 12, 24, and 36 hpi. Data are the results of three independent experiments (means ± SE). Significant differences are denoted by * *p* < 0.05, ** *p* < 0.01, and *** *p* < 0.001.

In order to identify whether bergamottin aroused IRF3 and NF-κB downstream cytokines, we detected the mRNA expression of some cytokines and interferon (IFN) in the absence or presence of bergamottin during PRRSV replication. We found that, compared with PRRSV-treated cells, Marc-145 cells processed with PRRSV and bergamottin showed an increase in IL-1β expression at 24 hpi (Figure 4A). Meanwhile, the expression of IL-6 was enhanced in the presence of bergamottin at 12 and 36 hpi (Figure 4B). Furthermore, IFN-α mRNA expression was up-regulated in the bergamottin-treated cells at 12 and 24 hpi (Figure 4C). Compared with cells infected with PRRSV alone, the expression of IFN-β was increased in the presence of bergamottin and significantly enhanced at 24 hpi (Figure 4D). Taken together, bergamottin activated IRF3 and NF-κB, leading to the increased expression of proinflammatory cytokines and type I interferon.

### 3.5. Bergamottin Inhibits Viral RNA Synthesis

**Figure 5:**
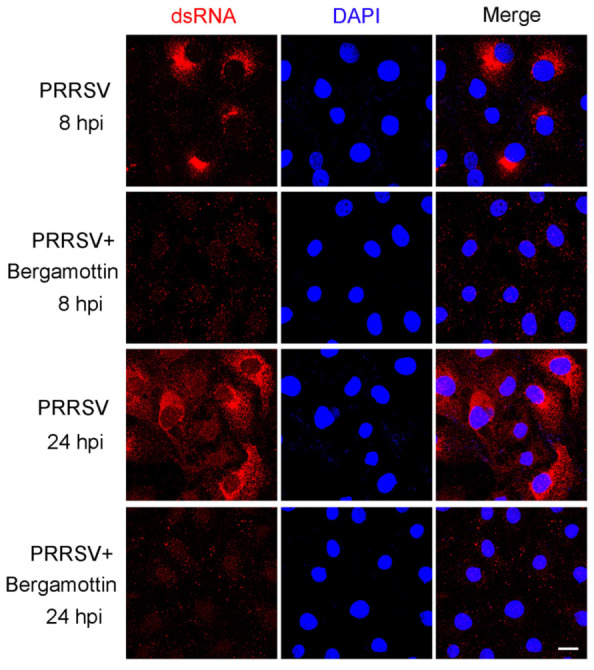
Bergamottin reduced viral dsRNA synthesis. Marc-145 were infected with PRRSV (MOI = 1) for 8 h or 24 h in the presence (25 μM) or absence of bergamottin. Cells were fixed and stained for dsRNA antibody, followed by an Alexa fluor 555-conjugated anti-rabbit IgG (red). Nuclei were counterstained with DAPI (blue). Bar = 25 μm.

PRRSV is known to produce dsRNA, which accumulates in the cell during the replication cycle [26]. We demonstrated that bergamottin inhibited PRRSV at the stage of replication, then we detected dsRNA expression in the absence or presence of bergamottin during PRRSV replication to identify whether it was due to the blocking of viral RNA synthesis. When Marc-145 cells were infected with PRRSV alone, dsRNA expression was increased at 8 hpi and increased to a maximum at 24 hpi accompanied by viral replication. In contrast, dsRNA production was significantly reduced in bergamottin-treated cells at 8 hpi, and it remained low up to 24 h after infection, which indicated bergamottin inhibited PRRSV replication in the early stage of replication by intercepting viral dsRNA formation (Figure 5). Collectively, bergamottin suppressed PRRSV RNA synthesis, thus inhibiting the PRRSV replication cycle.

### 3.6. Bergamottin Declines Most Nsp Expression and Completely Inhibited Nsp10

**Figure 6:**
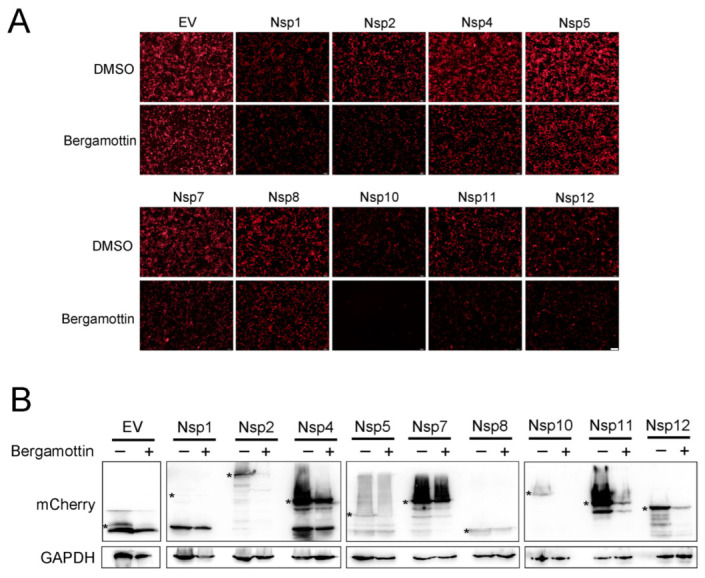
Bergamottin inhibited the expression of viral Nsps. mCherry-tagged viral Nsp plasmids (Nsp1, Nsp2, Nsp4, Nsp5, Nsp7, Nsp8, Nsp10, Nsp11, and Nsp12) were transfected into HEK293T cells for 6 h, respectively. Then, cells were treated with bergamottin (25 μM) for another 24 h. (**A**) Immunofluorescence analysis of Nsps-mCherry expression (Red); Bar = 200 μm. (**B**) Western blot analysis of Nsps-mCherry expression using anti-mCherry antibody, Asterisks mark the expressed mCherry-fusion proteins of viral Nsps.

The pp1a and pp1ab replicase polyproteins are expressed from ORF1a and ORF1b translation and then are processed into mature non-structural proteins, most of which assemble into a membrane-associated RTC [27]. RTC formation is important for PRRSV replication. It recognizes the 3′ of the genome to initiate minus-strand RNA synthesis, followed by genome replication and subgenomic mRNA synthesis. As the components of RTC, most Nsps are crucial for PRRSV replication. HEK293T cells were transfected with mCherry-tagged Nsp plasmids for 6 h and then treated with DMSO or bergamottin in the cells for another 24 h. Fluorescence analysis was used to identify viral Nsp expression. As shown in Figure 6A, bergamottin reduced the expression of Nsp2, Nsp7, Nsp10, Nsp11, and Nsp12. Remarkably, bergamottin could completely restrain Nsp10 expression. There was no obvious change in the fluorescence intensity of an empty vector, Nsp1, Nsp4, Nsp5, and Nsp8. Similarly, we analyzed Nsp expression using western blot. Compared with DMSO-treated cells, the expression of Nsp2, Nsp5, Nsp7, Nsp11, and Nsp10 was notably decreased in the cells with the presence of bergamottin. Nsp10 expression was also completely inhibited when cells were treated with bergamottin (Figure 6B). Taken together, bergamottin inhibited the protein expression of most Nsps (Nsp2, Nsp7, Nsp10, Nsp11, and Nsp12), thus interrupting RTC formation and viral RNA synthesis, and finally suppressed PRRSV replication.

## 4. Discussion

**Figure 7:**
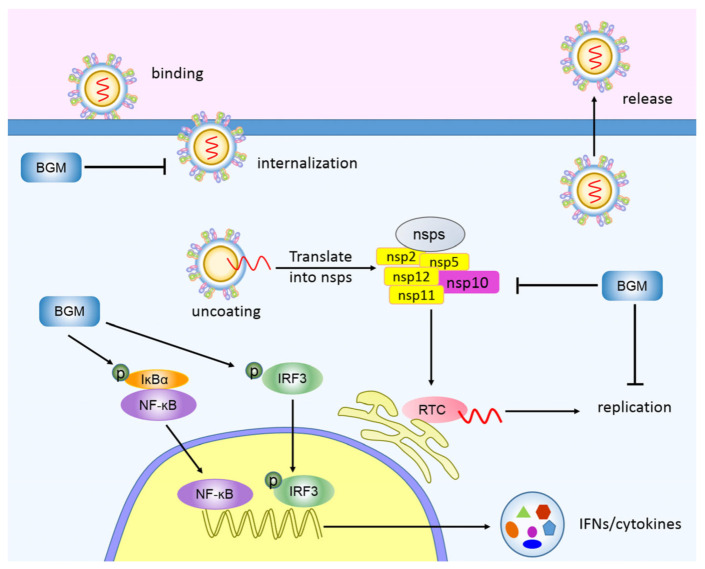
Schematic model of the inhibition of PRRSV by bergamottin. The life cycle of PRRSV includes binding, internalization, replication, and release. Bergamottin inhibited the stage of PRRSV internalization and replication. On the one hand, bergamottin reduced the expression of most Nsps and interrupted RTC formation and viral dsRNA synthesis, leading to the inhibition of PRRSV replication. On the other hand, bergamottin facilitated the activation of IRF3 and NF-κB signaling, promoting proinflammatory cytokines and interferon expression and suppressing PRRSV replication ultimately.

PRRSV is one of the most important pathogens in the pig industry, which causes huge economic losses worldwide. However, current PRRS vaccines cannot provide effective protection for the disease, and there are even no feasible drugs for the therapy [13,28]. In our study, we found a safe botanical compound called bergamottin, which is derived from citrus fruits and showed an inhibitory effect on PRRSV replication in vitro (Figure 1). Specifically, bergamottin inhibited PRRSV at the stages of internalization and replication. Bergamottin restrained most of the Nsp expression, especially Nsp10, and interrupted RTC formation, subsequently blocking viral RNA synthesis and ultimately suppressing PRRSV replication. Moreover, bergamottin activated NF-κB and IRF3 signaling pathways, leading to the increased expression of proinflammatory cytokines and interferon, which might be responsible for the inhibition of PRRSV replication (Figure 7).

In our study, bergamottin showed a nearly complete inhibition of the replication of the CHR6 strain, whereas it slightly inhibited other PRRSV strain replications (SD16, XJ17-5, and Li11) compared to the CHR6 strain (Figure 1D). Although they belong to PRRSV-2, the effect of bergamottin might be strain-specific to some extent. It is not surprising because different PRRSV strains obtain different sensitivities to cells and influence type I interferon response differently. Bergamottin has been reported to preferentially bind to ACE2 or degrade ACE2 mRNA and protein expression, thus preventing the interaction between Spike S protein and ACE2 [22]. Taking the attachment assay into consideration, there was no significant difference in viral binding in the presence of bergamottin (Figure 2B), which suggested that bergamottin did not affect PRRSV receptor expressions, such as CD169, Heparin sulfate, and CD163. In addition, it was reported that bergamottin could inhibit LASV infection by blocking LASV entry. Bergamottin interrupted LASV endocytic trafficking, thus hindering the transport of virions from the cell membrane to the cytoplasm [24]. In our study, we found that bergamottin also inhibited the stage of PRRSV entry (Figure 2C). It is known that CD151 and vimentin are involved in viral entry and are even associated with the complete inhibition of viral replication [29]. We speculate that bergamottin may competitively bind to these receptors or down-regulate their expression, then block viral entry. Moreover, whether bergamottin inhibited PRRSV via endocytosis or endosome trafficking needs further investigation. Bergamottin also inhibited PRRSV at the stage of replication (Figure 2D), and we further investigated the inhibitory mechanism in our study.

PRRSV replication is guaranteed by precise genetic and protein regulatory mechanisms. Initial PRRSV genome translation produces four polyproteins via programmed −1/−2 ribosomal frameshifting, including pp1a, pp1ab, pp1a-Nsp2TF, and pp1a-Nsp2N. These polyproteins are further processed into at least 14 non-structural proteins, including Nsp1α/β, Nsp2-Nsp6, Nsp7α/β, and Nsp8–Nsp12 [30]. These Nsps are assembled into a viral replication transcription complex (RTC) where RNA synthesis occurs. The RTC is involved in minus-strand RNA synthesis and produces both single-strand, full-length genome and subgenomic (sg) mRNAs. Structural proteins are expressed from the subset of sg mRNA [7,31]. Based on the PRRSV replication cycle, initial Nsp translation and RTC formation are the most important for PRRSV replication. To further investigate whether bergamottin inhibited PRRSV replication by blocking viral RNA synthesis, we detected dsRNA expression, which is an intermediate product of replication. We found viral dsRNA expression was notably decreased in the presence of bergamottin, suggesting bergamottin interrupted viral RNA synthesis (Figure 5). Furthermore, we observed bergamottin could reduce most of the Nsp expression, including Nsp2, Nsp7, Nsp10, Nsp11, and Nsp12. Among them, Nsp10 was completely inhibited when Nsp10 and bergamottin were both present (Figure 6). Nsp2 is an important component of RTC scaffolding, which is involved in the formation of RTC [4,32]. Nsp10 encodes the PRRSV helicase protein, which is critical to the generation of viral gRNA and sgRNA [7]. Nsp11 has a nidoviral uridylate-specific endoribonu-clease (NendoU) domain, which is required for viral replication, especially for sgRNA synthesis [8]. It was reported that Nsp12 recruits HSP70, thus facilitating PRRSV transcription and replication [33]. These Nsps are the components of RTC and are responsible for PRRSV replication. In our study, most of the Nsp expression declined in the presence of bergamottin or even failed to express, which hindered RTC formation, followed by RNA synthesis blocking, ultimately inhibiting PRRSV replication. Whether bergamottin inhibited Nsps by blocking the initial genome translation or post-translational degradation remains for further investigation.

Cytokines produced by innate immunity play an important role in the defense against viral infection and replication. Interferon and proinflammatory cytokines are particularly prominent in suppressing pathogens. RIG-I-like signaling and NF-κB signaling are the common pathways that produce type I interferon and proinflammatory cytokines [34]. In order to identify whether bergamottin had an influence on these pathways and cytokines, we detected phosphorylated IRF3 and NF-κB p65 nuclear translocations, which represent RIG-I and NF-κB pathways, respectively. We found that bergamottin activated IRF3 phosphorylation and NF-κB pathways (Figure 3). In addition, relevant cytokines, such as IL-1β, IL-6, IFN-α, and IFN-β, were enhanced to some extent (Figure 4). These data suggested bergamottin triggers IFN and inflammatory responses mainly through IRF3 and NF-κB activation. We can also investigate if there is another pathway or mechanism that bergamottin inhibits PRRSV in future work.

We identified bergamottin’s anti-PRRSV effect in vitro and its underlying mechanism. However, whether bergamottin has therapeutic effects on PRRSV-infected pigs remains unclear. Firstly, the innocuousness of bergamottin at the organismic level seems to be still rather controversial. Secondly, the dose to control PRRSV replication in vivo and the toxic dose to the animals are uncertain. Thirdly, because of the poor economic benefit and many unknowns at the organismal level, it is hard to apply antivirals in production species and produce even a little effect.

In conclusion, we demonstrated that bergamottin, a bioactive component of bergamot, inhibited PRRSV replication. On the one hand, bergamottin promoted the activation of IRF3 and NF-κB pathways, thus facilitating the expression of IFN and proinflammatory cytokines and inhibiting PRRSV replication ultimately. On the other hand, bergamottin decreased most of the Nsp expression and blocked viral RNA synthesis, leading to the inhibition of PRRSV replication. Our data evaluated the inhibitory effects of bergamottin on PRRSV replication and provided a new way to prevent PRRSV replication in vitro.

## Data Availability

All the data generated during the current study are included in the manuscript. Additional data related to this article may be requested from the corresponding authors.
